# NMR Structure of Lipoprotein YxeF from *Bacillus subtilis* Reveals a Calycin Fold and Distant Homology with the Lipocalin Blc from *Escherichia coli*

**DOI:** 10.1371/journal.pone.0037404

**Published:** 2012-06-05

**Authors:** Yibing Wu, Marco Punta, Rong Xiao, Thomas B. Acton, Bharathwaj Sathyamoorthy, Fabian Dey, Markus Fischer, Arne Skerra, Burkhard Rost, Gaetano T. Montelione, Thomas Szyperski

**Affiliations:** 1 Department of Chemistry, State University of New York at Buffalo, Buffalo, New York, United States of America; 2 Department of Computer Science and Institute for Advanced Study, Technical University of Munich, Munich, Germany; 3 Center of Advanced Biotechnology and Medicine, Department of Molecular Biology and Biochemistry, Robert Wood Johnson Medical School, The State University of New Jersey, Piscataway, New Jersey, United States of America; 4 Howard Hughes Medical Institute, Department of Biochemistry and Molecular Biophysics, Center for Computational Biology and Bioinformatics, Columbia University, New York, New York, United States of America; 5 Munich Center for Integrated Protein Science, CIPS-M, and Lehrstuhl für Biologische Chemie, Technische Universität München, Freising-Weihenstephan, Germany; 6 Northeast Structural Genomics Consortium; Griffith University, Australia

## Abstract

The soluble monomeric domain of lipoprotein YxeF from the Gram positive bacterium *B. subtilis* was selected by the Northeast Structural Genomics Consortium (NESG) as a target of a biomedical theme project focusing on the structure determination of the soluble domains of bacterial lipoproteins. The solution NMR structure of YxeF reveals a calycin fold and distant homology with the lipocalin Blc from the Gram-negative bacterium *E.coli*. In particular, the characteristic β-barrel, which is open to the solvent at one end, is extremely well conserved in YxeF with respect to Blc. The identification of YxeF as the first lipocalin homologue occurring in a Gram-positive bacterium suggests that lipocalins emerged before the evolutionary divergence of Gram positive and Gram negative bacteria. Since YxeF is devoid of the α-helix that packs in all lipocalins with known structure against the β-barrel to form a second hydrophobic core, we propose to introduce a new lipocalin sub-family named ‘slim lipocalins’, with YxeF and the other members of Pfam family PF11631 to which YxeF belongs constituting the first representatives. The results presented here exemplify the impact of structural genomics to enhance our understanding of biology and to generate new biological hypotheses.

## Introduction

The lipoprotein YxeF from *Bacillus subtilis* was selected by the Northeast Structural Genomics Consortium (NESG; http://www.nesg.org) as a target (gi|85674274, SwissProt/TrEMBL ID YXEF_BACSU, access number P54945, NESG target ID SR500A) of a biomedical theme project focusing on the structure determination of the soluble domains of bacterial lipoproteins [1], [2]. YxeF exhibits no significant sequence similarity with any protein with known three-dimensional structure and is one of only eight members forming Pfam [3] family PF11631 for which no functional annotation is available (Pfam 26.0 release). All members of the family are from the genus *Bacillus* and present high sequence similarity to YxeF, with A7ZAF5 and E1UTS8 from *B. amyloliquefaciens* being the most distant homologues (61% sequence identity to YxeF over 129 residues).

Bacterial lipoproteins represent a class of secreted, membrane-anchored proteins that are conserved throughout bacteria and play critical roles in a wide range of biological processes, including bacterial pathogenesis and host immune response [1]. They contain a conserved N-terminal type II signal peptide also known as the ‘lipobox’, which is immediately followed by an invariant Cys residue. After cleavage of the signal peptide, the lipoprotein is anchored into the bacterial membrane *via* a diacylglycerol moiety forming a thioether linkage to the Cys side chain as well as a fatty acid moiety coupled to the N-terminus.

In general, lipobox sequences exhibit the consensus sequence [Leu/Val/Ile]-[Ala/Ser/Thr/Val/Ile]-[Gly/Ala/Ser]-[Cys] (Val-Ser-Gly-Cys in YxeF). The residue following the Cys is important for localization of the lipoprotein [1]. In Gram-negative bacteria the lipoprotein is usually anchored in the inner membrane if Cys is followed by Asp, whereas otherwise it is anchored in the inner leaflet of the outer membrane [2]. Although Cys is followed by Gln in YxeF, it is supposedly anchored in the only lipid membrane of the Gram-positive *Bacillus subtilis* cell. Here we report the high-quality NMR solution structure of the soluble domain of protein YxeF comprising residues 19–144, along with a structural bioinformatics analysis to classify its structure and to gain new insights into its evolutionary origin.

## Results and Discussion

### NMR Structure of the Soluble Domain of Lipoprotein YxeF

**Figure 1:**
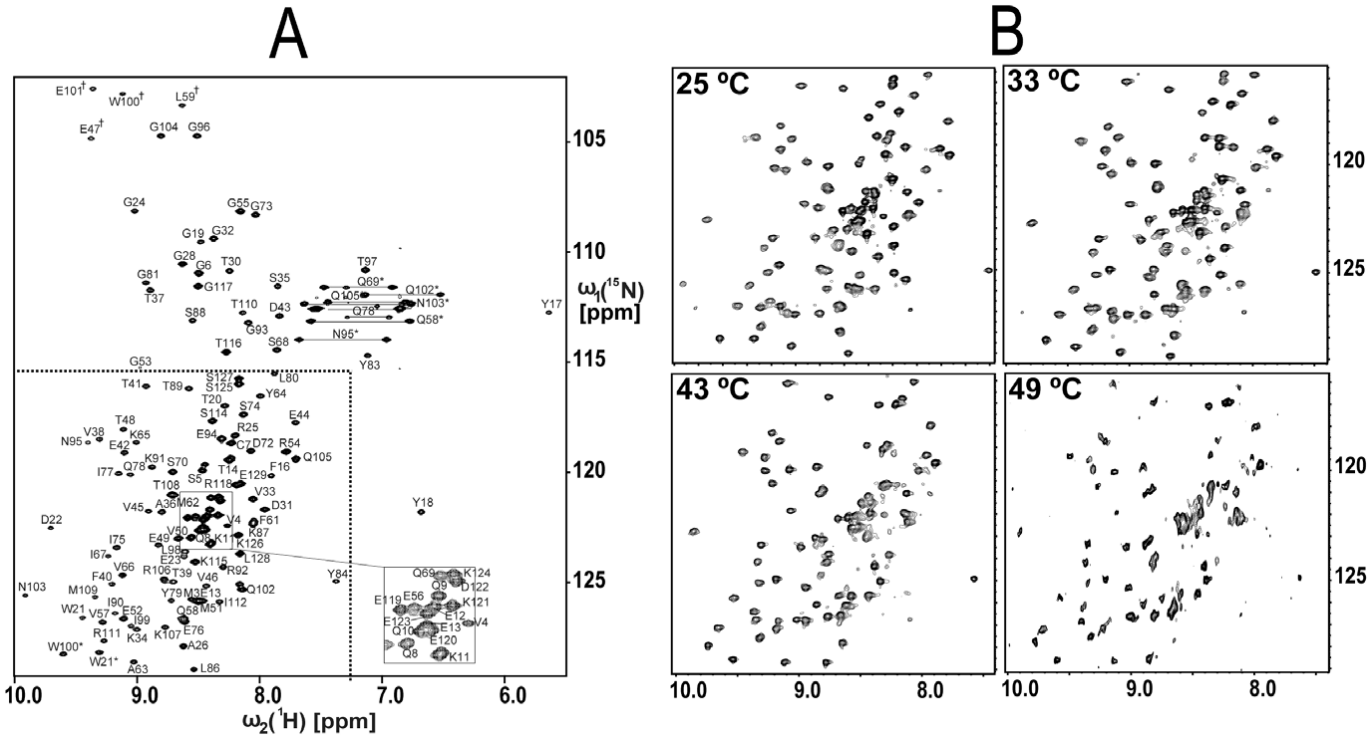
2D [^15^N,^1^H] HSQC spectra of lipoprotein YxeF. (A) Spectrum recorded for the sample used for NMR structure determination at 750 MHz ^1^H resonance frequency. Resonance assignments are indicated using the one-letter amino acid code. Signals arising from side chains (Asn H^δ2^/N^δ2^, Gln H^ε2^/N^ε2^, Arg H^ε^/N^ε^ and Trp H^ε1^/N^ε1^) are labeled with (*) and folded signals are designated with (†) next to the residue number. Signals arising from the His purification tag were not sequence specifically assigned. The spectral region indicated by dotted lines comprises most of the signals arising from the β-barrel (Figure 2) and is displayed for the spectra shown in (B). Those were recorded at different temperatures at 500 MHz ^1^H resonance frequency (see text).

**Figure 2:**
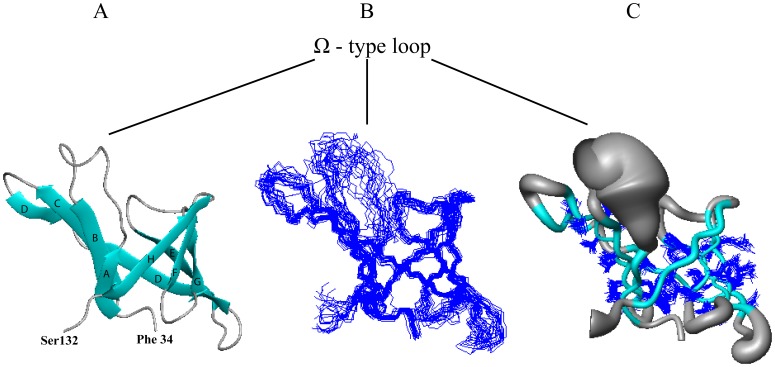
NMR structure of the soluble domain of lipoprotein YxeF. Only residues 34–132 are shown because the terminal polypeptide segments are flexibly disordered in solution. The N-terminal residue Phe 34 and the C-terminal residue Ser 132 are labeled. Lines point at the Ω-type loop, which connects β-strands A and B and is poorly defined in the NMR structure. (A) Ribbon drawing of the first conformer of PDB entry 2JOZ with β-strands being depicted in cyan. (B) Superposition of the 20 conformers representing the NMR solution structure obtained after superposition of the backbone heavy atoms (N, C^α^ and C’) of the β-strands. (C) “Sausage” representation of backbone and best-defined side chains: a spline function was drawn through the mean positions of C^α^ atoms with the thickness being proportional to their mean global displacement in the 20 conformers after superposition as in B). The figure was generated using the program MOLMOL [59].

**Figure 3:**
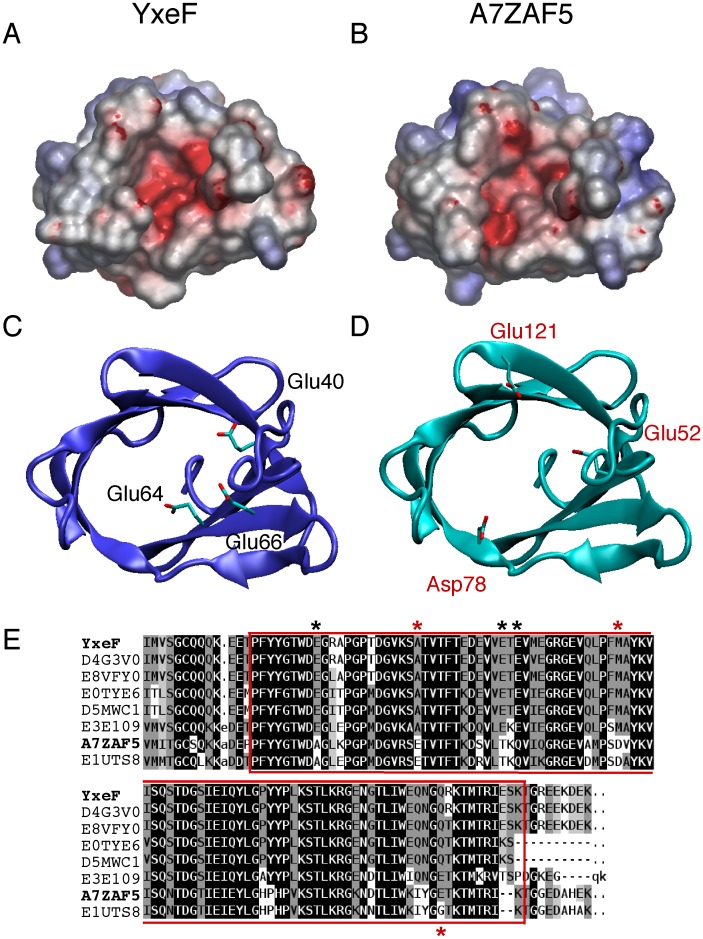
Comparison of *B. subtilis* YxeF NMR structure and *B. amyloliquefaciens* A7ZAF5 homology model. Surface electrostatic potential calculated for (A) the YxeF NMR structure (first conformer of ensemble deposited in the PDB) and (B) the homology model of A7ZAF5 by using the program GRASP [56] accessed through the protein function annotation server MarkUs [55]. The homology model was calculated using the SWISS-MODEL server in alignment mode [60], [61] and Verify3D [63], Procheck [64] and ProsaII [65] all atom z-scores (-1.12, −3.43 and −1.61, respectively) were obtained using the PSVS server [66] and are indicative of a good quality model. In (C) and (D), ribbon drawings are shown for the structures of YxeF and A7ZAF5 in the same orientation, that is, viewed on the open end of the β-barrels. The acidic residues giving rise to the negative potential inside the cavities are depicted in licorice representation and are labeled (black for YxeF, red for A7ZAF5). (E) Pfam multiple alignment of the sequences of all members of PF11631. Except for YxeF (P54945), the sequences are labeled with their UniProt [25] IDs (D4G3V0, E8VFY0, E0TYE6, D5MWC1, E3E109, A7ZAF5, E1UTS8). Amino acid background colors reflect average similarity inferred from the Blosum62 matrix, ranging from ‘most conserved’ (black) to ‘least conserved’ (white). YxeF and A7ZAF5 are highlighted in bold on the left and the region of the alignment used for building the comparative model of A7ZAF5 from the YxeF structure is enclosed by red boxes. The acidic residues labeled in (C) and (D) are marked with black (YxeF) and red (A7ZAF5) asterisks, respectively, above or below the alignment.

A high-quality NMR structure of the soluble domain (comprising residues 19–144) of lipoprotein YxeF was obtained by multidimensional NMR spectroscopy [Figures 1A, 2; Table 1; PDB (protein data bank) accession number 2JOZ]. For simplicity, we henceforth refer to the structure of this domain as to the ‘YxeF structure’. YxeF contains nine β-strands (A to D and D’ to H) comprising residues 36–40, 52–57, 61–68, 72–75, 79–86, 91–97, 104–109, 114–119, and 122–129, respectively (Figure 2; strand assignment according to STRIDE [4]). Since strands D and D’ point into the same direction and are connected by a short coil region in a mostly extended conformation (comprising Pro 78), we will refer to this entire polypeptide segment as strand D (Figure 2). All β-strands are then arranged in anti-parallel fashion forming a +1 up-and-down β-barrel and are connected by seven loops L1 to L7. The β-barrel is closed on one side (loops L2, L4, L6), primarily by dense side chain packing of a number of hydrophobic and aromatic residues, including Phe 33, Tyr 34, Tyr 35, Trp 38 located immediately upstream of or on β-strand A, and additionally Tyr 81 and Leu 115. On the other side the β-barrel is open to the solvent (loops L1, L3, L5, L7) and lines a cavity with overall negative charge, predominantly due to the presence of Glu 40 on β-strand A, and Glu 64 and Glu 66 on β-strand C (Figure 3A,C).

**Table 1:** Statistics of YxeF(19–144) NMR Structure.

Completeness of stereo-specific assignments^a^ [%]	
^ α^CH_2_ of Gly	8 (1/13)
^ β^CH_2_	20 (16/80)
Val and Leu methyl groups	79 (11/14)
Conformationally-restricting distance constraints	
Intraresidue [*i* = *j*]	462
Sequential [|*i – j*| = 1]	556
Medium Range [1< |*i – j*| <5]	148
Long Range [|*i – j*| >5]	662
Total	1828
Dihedral angle constraints (φ/ψ)	(49/49)
Number of constraints per residue	15.2
Number of long-range distance constraints per residue	5.2
CYANA target function [Å^2^]	1.14+0.15
Average number of distance constraints violations per CYANA conformer	
0.2–0.5 Å	0
>0.5 Å	0
Average number of dihedral-angle constraint violations per CYANA conformer >5°	1.0
Average r.m.s.d. to the mean CNS coordinates [Å]	
Regular secondary structure elements^b^, backbone heavy atoms	0.51±0.13
Regular secondary structure elements^b^, all heavy atoms	0.92±0.12
Ordered residues^c^, backbone heavy atoms	0.81±0.14
Ordered residues^c^, all heavy atoms	1.26±0.15
Heavy atoms of molecular core including best-defined side chains^d^	0.55±0.09
PROCHECK^63^ G-factors raw score (φ and ψ/all dihedral angles)^c^	−0.51/−0.38
PROCHECK^63^ G-factors Z-score (φ and ψ/all dihedral angles)^c^	−1.69/−2.25
MOLPROBITY^64^ clash score (raw/Z-score)^c^	17.34/−1.45
PRF R/P/DP scores^53^ [%]	0.99/0.87/0.85
Ramachandran plot summary c [%]	
most favored regions	93.0
Additionally allowed regions	6.5
generously allowed regions	0.2
disallowed regions	0.2

a Relative to pairs with non-degenerate chemical shifts.

b Residues 37–40, 53–58, 62–69, 73–76, 80–86, 92–98, 105–110, 116–120, and 123–128.

c Residues 33–40, 45–48, 51–87, 93–96, 99–101, 104–111, 115–131.

d Backbone and side-chain heavy atoms of residues 37–39, 54–59, 63–64, 66, 68, 75, 77, 81–82, 84–86, 93–96, 98, 105–108, 115–118. Best-defined side chains are those exhibiting a displacement of less than 1 Å for their side chain heavy atoms after superposition of the β-strands for minimal r.m.s.d.

### Current Classification of YxeF Structure in the CATH, SCOP and Pfam Databases

**Figure 4:**
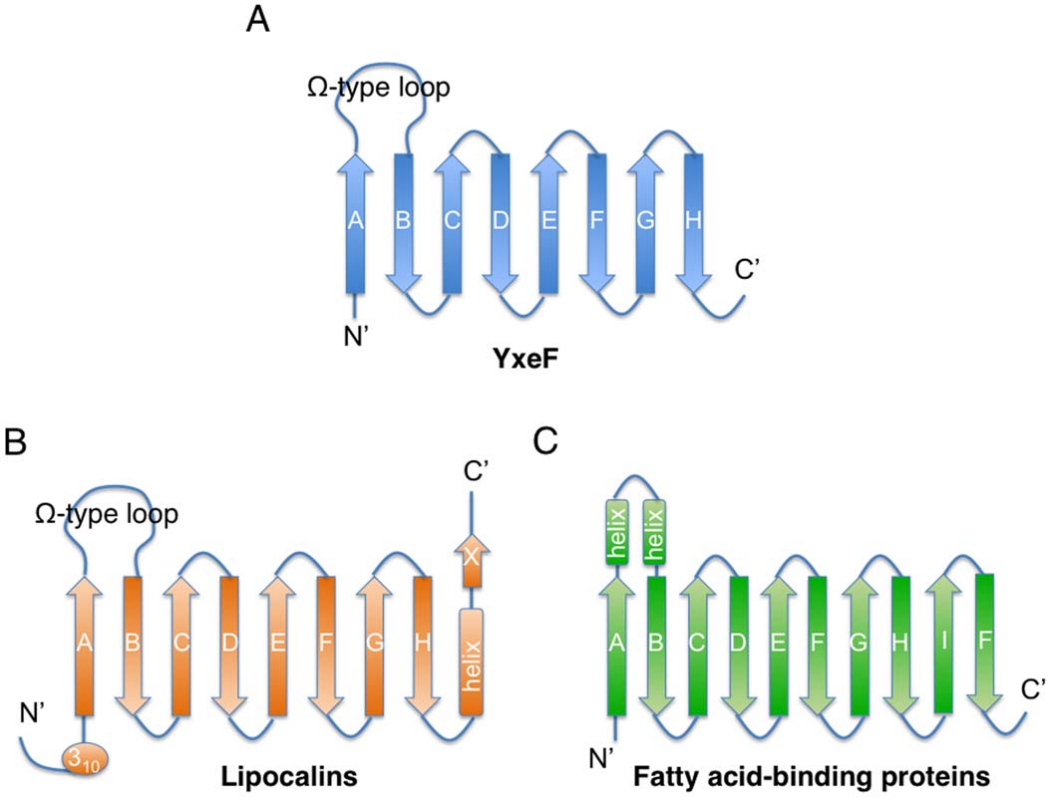
Schematic representation of secondary structure element topologies. (A) YxeF, (B) lipocalins and (C) fatty acid-binding proteins. β-strands are represented by arrows, α-helices by rectangles, and 3_10_-helices by ellipses. N- and C-termini are indicated as N and C respectively, and the ‘Ω-type’ loop L1 shared by YxeF and lipocalins is labeled.

Inspection of the YxeF structure (Figure 2) shows that it resembles β-barrel proteins belonging to the ‘calycin *super*family’ which includes lipocalins, fatty acid binding proteins, triabin, avidins/streptavidins and a class of metalloprotease inhibitors. All calycins contain a calyx-like β-barrel characterized by a +1 up-and-down topology (Figure 4), with triabin being the only exception due to a β-strand swap, and fatty acid-binding proteins featuring two additional β-strands in the barrel with respect to other calycins (*i.e.*, 10-stranded instead of 8-stranded) [5], [6]. The β-barrels structurally characterizing calycins are open to the solvent on one side and often harbor a ligand-binding site [6], [7].

Accordingly, our YxeF structure (Figure 2) has been incorporated in the CATH (class architecture topology homologous superfamily) and SCOP (structurally classification of proteins) databases [8], [9]. In CATH, it is part of the Homology sub-level 2.40.128.20 within the ‘lipocalin’ Topology. This Homology sub-level incorporates lipocalins, fatty acid binding proteins and triabin, while the ‘lipocalin’ Topology includes all other calycins together with additional members such as some outer membrane proteins. In SCOP, YxeF is assigned to the ‘retinol binding protein-like’ family, containing all lipocalins of known structure. This family is found within the ‘lipocalin’ SCOP *super*family further including fatty acid binding proteins and triabin. Avidin/streptavidin and metalloprotease inhibitors are instead assigned to a different SCOP fold (*i.e.*, ‘streptavidin-like’). Finally, in the Pfam sequence database lipocalins are grouped with fatty acid-binding proteins in several families within the ‘calycin superfamily’ clan [10], which additionally includes triabin. Avidins/streptavidins and metalloprotease inhibitors are not considered to be part of the ‘calycin superfamily’. These classifications are (i) based on both sequence and structure comparisons, (ii) rely, at least to some degree, on manual curation, and (iii) favor the hypothesis that an evolutionary link exists between lipocalins, fatty acid binding proteins and triabin. They leave, however, the tetrameric avidins/streptavidins and some metalloprotease inhibitors in *limbo* with respect to their relationship to the other proteins alluded to above.

SCOP further identifies lipocalins as a sub-group of more closely related proteins and places YxeF among them. Lipocalins are extracellular (sometimes membrane anchored) proteins known to generally transport and store small, largely hydrophobic compounds within a ligand pocket surrounded by four loops at the open end of the β-barrel [5], [11]. Despite sharing with lipocalins the same β-barrel topology YxeF lacks a C-terminal α-helix (Figure 4A,B) which, in all lipocalins with known structure, packs against one side of the β-barrel. This observation raises the question of whether and how YxeF is evolutionary related to lipocalins. One of the key challenges associated with classifying calycin−/lipocalin-like proteins is their typically very low (*i.e.*, insignificant) sequence identity, so that quite often homology cannot be inferred from sequence alone [5], [6]. Furthermore, the manifold of known eight stranded β-barrels appears to form what has been named a structural ‘quasi-continuum’ [12]. This greatly impedes the identification of boundaries between divergent and convergent evolutionary links. In the following, we present a structural bioinformatics analysis aimed at resolving the YxeF structure classification and elucidating YxeF’s evolutionary origin.

### YxeF Structure Belongs to the Calycin Superfamiliy

**Figure 5:**
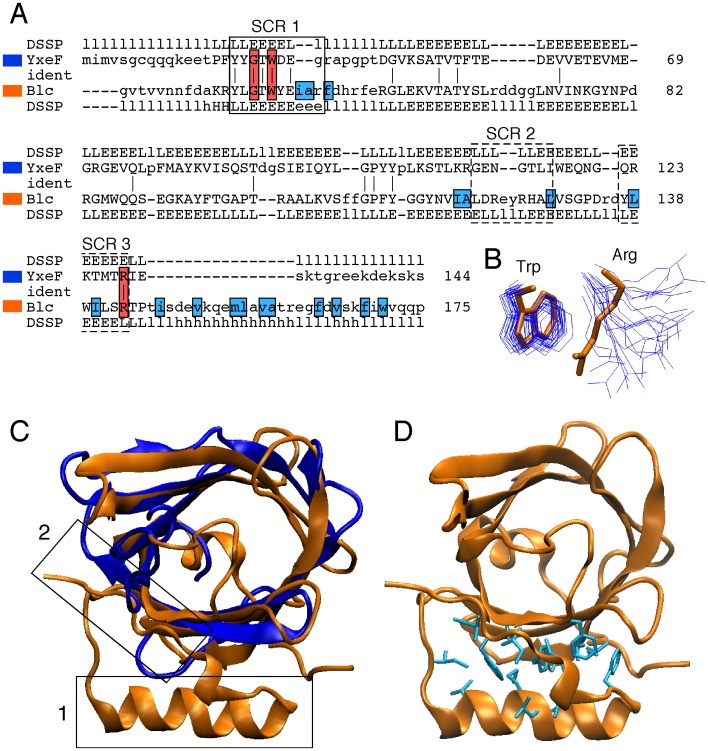
Comparison of YxeF NMR structure (PDB ID 2JOZ, coded in blue) and Blc X-ray crystal structure (PDB ID 3MBT, orange). (A) Structure-based sequence alignment between YxeF and Blc obtained with the program DALI [16]. The three structurally conserved regions (SCR1-3) typically found in lipocalins (see text) are boxed (continuous line for SCR1, which appears to be conserved in YxeF; dashed line for SCR2 and SCR3). Conserved residues being part of the calycin signature motif resulting in an interaction between Gly 36-X-Trp 38 in SCR1 and Arg 128 in SCR3 (see text) are highlighted using red boxes. Residues being part of the second hydrophobic core of Blc [see also (D] are highlighted using cyan boxes. (B) Superposition of the Trp and Arg residues being part of the calycin Gly-X-Trp and Arg motif in Blc (licorice representation, orange) and YxeF (line representation, all NMR conformers, blue). The superposition is obtained after superposition of the X-ray structure of Blc with each conformer of the NMR solution structure of YxeF (residues 32–132). (C) Structural superposition generated by the program DALI viewed from the open end of the β-barrels (for YxeF residues 32–132 were considered). In Blc, box 1 identifies the C-terminally located α-helix and box 2 the C-terminal β-strand, which are packed against the outside of the β-barrel and thereby form a second hydrophobic core (see D). (D) Ribbon drawing of the Blc structure with licorice representation of hydrophobic residues (in cyan) located in the C-terminal α-helix and on the outside of the β-barrel forming a second hydrophobic core [see also (C)].

Calycins feature a conserved Gly-X-Trp/Arg signature motif (Figure 5), in which the Arg side chain is located on strand H, interacts with the Trp side chain located on strand A and also forms hydrogen bonds with the backbone carbonyl groups of some other N-terminal residues [5], [11], [13]. Consistently, this motif has been shown to be important for protein stability in the retinol-binding protein, a prototypic member of the lipocalin family [14], [15]. In YxeF, the motif is entirely conserved (Gly 36, Trp38 and Arg 128), although the conformation of the side chain of Arg 128 is rather poorly defined in the NMR structure (Figure 5B). Conservation of the calycin signature motif and of the β-barrel topology (Figure 4) renders straightforward the classification of YxeF as a ‘calycin’.

### DALI-based Search for Similar Structures

A search of the PDB using the program DALI [16] for proteins which are structurally similar to YxeF yielded more than 700 significant hits (152 when considering PDB90 or a set of PDB proteins redundancy reduced at 90% sequence identity). These hits span a quasi-continuum of Z-scores between 7.7 (top hit) and 2.1 (taken as a lower limit of significance). Among the structurally similar proteins are calycins from all groups alluded to above. Top hits include the cysteine protease inhibitor staphostatin B (Z-score  = 7.7), several 10-stranded fatty acid binding proteins (top Z-score  = 7.5), the C-terminal domain of a self-compartmentalizing protease Pab87 from *Pyrococcus abyssi* (Z-score  = 7.2) which has been claimed [17] to be the first domain with lipocalin-like architecture from Archaea, and several lipocalins (top Z-score 6.9). Staphostatin B is classified in SCOP as having a ‘Streptavidin-like’ fold but high structural similarity to lipocalins was recognized previously [18]. It should be noted, however, that in spite of the high Z-score returned by overall structure superposition, topology of strand A to C in staphostatin’s β-barrel is very different with respect to the one observed in both streptavidins and lipocalins. Other calycin structures result in lower DALI Z-scores (avidin’s Z-score is 5.5).

Overall structural similarity identified with DALI thus seems to clearly indicate evolutionary relatedness between YxeF, fatty-acid binding proteins and lipocalins. Two structural features shared by YxeF and lipocalins, however, strongly suggest that YxeF is closer to the latter than to fatty-acid binding proteins (Figure 4): (i) the 8 strands forming the β-barrel (*versus* 10 in fatty acid-binding proteins), and (ii) the presence of an ‘Ω-type loop’ connecting β-strands A and B (in fatty-acid binding proteins two short α-helices are inserted between strands A and B). Notably, the Ω-type loop that often acts as a flexible lid for the open end of the β-barrel in lipocalins appears to be disordered in YxeF (Figure 2). On the other hand, as mentioned above, YxeF lacks two structural features conserved in the lipocalins (Figure 4): an N-terminal 3_10_-helix, which is sometimes replaced by a longer α-helix [19], [20], and a C-terminally located α-helix followed by an additional β-strand [21], [22]. With respect to the first difference, several aromatic residues located in YxeF in the polypeptide segment preceding β-strand A (*i.e.*, Phe 33, Tyr 34, Tyr 35) play a very similar structural role as the forming the 3_10_-helix in lipocalins, that is, they contribute to occlude the bottom of the β-barrel. Thus, solely the absence of the C-terminal α-helix and β-strand remain as a stark structural difference when comparing the structure of YxeF with known lipocalin structures.

### YxeF and Lipocalin Blc from *E. coli* are Distant Homologues

To further refine our structural analysis, we compared in detail the structure of YxeF with that of lipoprotein Blc from *E. coli* (Figure 5), the only bacterial lipocalin for which an atomic-resolution structure is currently available (PDB ID: 3MBT) [23]. It has been suggested that lipocalins can be grouped into ‘kernel’ or ‘outlier’ lipocalins [11] depending on the presence or absence of three so-called ‘structurally conserved regions’ (SCRs) which correlate to some degree with sequence conservation (Figure 5A). The most conserved SCR1 comprises the N-terminal Gly-X-Trp segment of the calycin Gly-X-Trp/Arg signature motif located on β-strand A, while SCR3 (formed primarily by residues found on β-strand H) contains the Arg residue as part of the same motif. SCR2, instead, spans the region between the termini of β-strands F and G including the loop L6 connecting the two β-strands. Because the SCR2 and SCR3 sequence motifs are not conserved in Blc, it was initially classified as an ‘outlier lipocalin’ [24]. However, this sequence-based classification does not appear to be well justified when considering that the X-ray structure of Blc revealed a remarkably close structural similarity with ‘kernel’ lipocalins [23].

**Figure 6:**
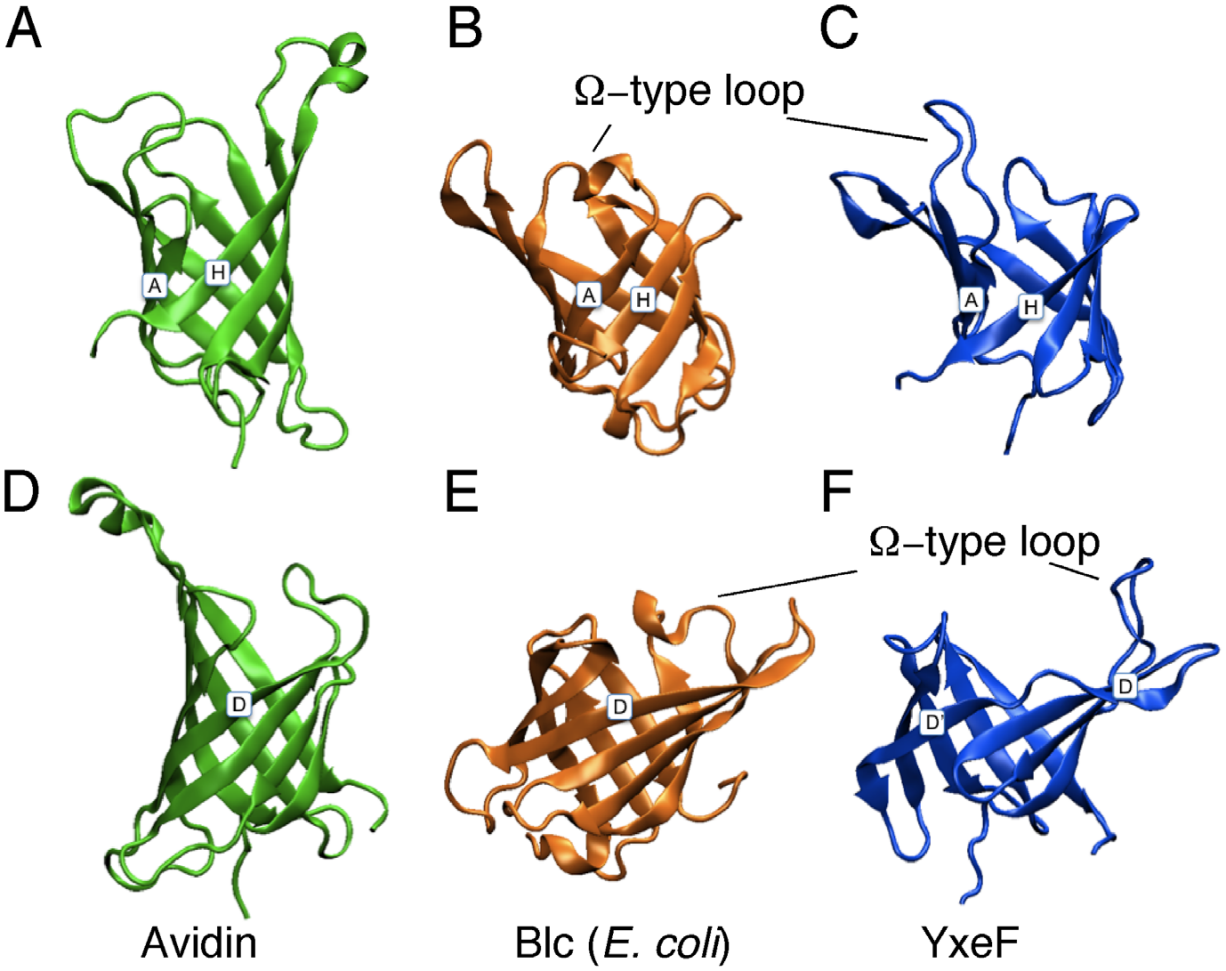
Comparison of β-barrels. Ribbon drawings of β-barrels of avidin (PDB ID 1AVD, green) in (A) and, after rotation by 180°, in (D); bacterial lipocalin Blc from *E. coli* (PDB ID 3MBT, orange) in (B) and (E); YxeF in (C) and (F) (PDB ID 2JOZ, blue). For clarity, the disordered terminal polypeptide segments of YxeF, as well as the corresponding segments in avidin and Blc, are not shown. In (A)–(C), β-strands A and H are labeled, while in (D)–(F) β-strand D is indicated.

Like YxeF, Blc is monomeric and does not contain disulfide bridges, which are frequently found in other lipocalins. While the overall structural alignment of YxeF and Blc results in a highly significant, but not the highest DALI Z-score [Z-score  = 6.9; 2.7 Å r.m.s.d. (root mean square deviation) for superposition of the C^α^ atoms of 88 aligned residues exhibiting 17% sequence identity], the structural similarity of the two β-barrels (48 residues) is truly striking: they superimpose with a backbone r.m.s.d. of only 1.8 Å (Figure 5). Indeed, the structural alignment of only the β-barrels yields the highest Z-score for Blc among a selection of full structure alignment top hits (Table 2; note that in these comparisons we consider conformer 1 of the ensemble representing the YxeF solution structure 2JOZ). The corresponding values calculated for avidin, which contains a β-barrel of evidently different shape (Figure 6), are also provided in Table 2 for comparison. The only minor structural difference between the YxeF and Blc β-barrels, possibly reflecting different ligand specificities, relates to β-strand A at the base of the Ω-type loop. This strand is shorter in YxeF where it creates a small V-shaped aperture on the side of the β-barrel (Figure 6).

**Table 2:** Comparison of β-barrels occurring in selected structures^a^ yielding top DALI Z-scores after full structure alignment with YxeF.

Structure compared withYxeF structure	β-barrel only				DALI Z-score forentire structure
	DALI Z-score	r.m.s.d.(Å)	Number of residues	Sequence identity	
1Y4H(C)	4.4	1.7	40	13%	7.7
3D95(B)	4.2	2.7	49	18%	7.5
1JJJ(A)	4.2	2.9	50	12%	7.4
2QMI_C(B)	3.9	1.9	40	8%	7.2
3AKM(A)	4.2	3.0	50	12%	7.1
1P6P	4.5	2.9	49	8%	7.1
**Blc**	**5.7**	**1.8**	**48**	**15%**	**6.9**
2CAM(A)	3.8	2.3	46	13%	5.3

a Structures were selected among those with the top DALI Z-score (see text) when aligned with the entire folded YxeF domain (residues 32–132), and they are ranked according to the Z-score from that comparison (right-most column). The location of the β-strands forming the β-barrels were identified using the program STRIDE^4^ and pairwise structural comparisons of the β-barrels was again performed using the program DALI.^16,58^ R.m.s.d. values were calculated after superposition of the C^α^ atoms. Structures of proteins other than *E. coli* Blc (seventh row, highlighted in bold) are designated by their PDB IDs: 1Y4H (chain C) is staphostatin B; 3D95 (chain B) is the cellular retinoic acid-binding protein II; 1JJJ (chain A) is the human epidermal-type fatty acid-binding protein; 2QMI_C (chain B) is the C-terminal domain of protease Pab87; 3AKM (chain A) is the human intestinal fatty acid binding protein; 1P6P is the toad liver basic fatty acid-binding protein. For comparison, the values for avidin (PDB ID 2CAM, chain A) are also provided (bottom row; see also Figure 6).

As indicated above, the C-terminally located α-helix characteristic of lipocalins is not present in YxeF (Figure 4). In Blc, this α-helix packs against the outside of the β-barrel, primarily against β-strands G and H (Figure 5C). As a result, a second hydrophobic core is formed, adding to the one found in the lower, closed part of the β-barrel itself (Figure 5D). In YxeF, the corresponding C-terminally located polypeptide segment is highly polar and flexibly disordered in solution. The absence of hydrophobic residues in this segment and also on the exterior of β-strands G and H apparently prevents the formation of a second hydrophobic core. Since this core has been shown to be important for the stability of lipocalins, and Blc exhibits a comparably low melting temperature of ∼45°C even with this second core, the stability of the well-defined fold of YxeF evidenced by our high-quality structure (Figure 2) is somewhat unexpected. Intriguingly, although the onset of protein precipitation at higher temperature prevented us from accurately determining a heat denaturation ‘melting’ temperature, the inspection of 2D [^15^N,^1^H] HSQC (heteronuclear single quantum coherence) spectra recorded up to ∼50°C revealed that YxeF’s β-barrel is intact even at such elevated temperatures (Figure 1B).

Taken together, in spite of the lack of the C-terminally located α-helix, the strong structural similarity of the β-barrels of YxeF and Blc (Table 2), together with a remarkably similar relative spatial orientation of the Trp and Arg residues of the Gly-X-Trp/Arg signature motif (Figure 5B), reveals that YxeF and lipocalin Blc are distant homologues. This is consistent with the fact that both YxeF and Blc are secretory lipoproteins, a characteristic common to most predicted lipocalins in Gram-negative Bacteria [24].

### Homology Model of Protein A7ZAF5: Insights into Putative Ligand Binding

The only proteins known to share significant sequence identity with YxeF are found in the *Bacillus* genus, *e.g.*, D5MWC1 from *B. subtilis* strain ATCC 6633, E3E109 form *B. atrophaeus* strain 1942, and the somewhat more distant homolog A7ZAF5 from *B. amyloliquefaciens* (61% identity; member of PF11631; see Figure 3E for a multiple sequence alignment of all members). In D5MWC1 and E3E109, the three glutamate residues that confer a negative charge distribution to YxeF’s putative ligand binding site (Figure 3A,C) are conserved. Despite the high overall sequence similarity, these glutamates are absent in A7ZAF5. Interestingly, however, in a homology model calculated for the soluble domain of A7ZAF5 based on conformer 1 of the YxeF NMR structure (2JOZ), it appears that other negatively charged residues, *i.e.*, Glu 52, Asp 78, Glu 121 (residue numbers as in the UniProt [25] sequence of A7ZAF5) create a similar surface charge distribution within the corresponding cavity of A7ZAF5 (Figure 3B,D,E; note that Glu 121 is replaced by Gly in E1UTS8, while additionally conserved in E3E109). Considering the robustness of homology modeling at >60% pairwise sequence identity and the absence of any gaps in the alignment, this finding suggests that these proteins may function by binding yet to be identified ligands that share similar electrostatic properties.

### Evolutionary Origin of Lipocalins and Protein YxeF

Lipocalins are a functionally diverse group of proteins that usually bind small hydrophobic molecules. Bacterial lipocalins, in particular, have been proposed to be implicated in biogenesis of the outer membrane of Gram-negative bacteria based on three findings: (i) Blc from *E. coli* has been shown to bind fatty acids and (lyso)-phospholipids [26], suggesting its participation in lipid metabolism [26], (ii) no lipocalin had so far been identified in microorganisms that lack an outer lipid membrane, that is, Gram-positive bacteria or Archaea, and (iii) some integral outer membrane proteins exhibit significant structural similarity with lipocalins [21], [27].

Our finding that YxeF and Blc are distant homologues suggests to classify YxeF (and thus all currently known members of PF11631 exhibiting high sequence similarity; Figure 3E) as a hitherto unknown type of lipocalin constituting a new sub-family characterized by its distinct ‘β-barrel only’ architecture. We suggest to name this new sub-family 'slim lipocalins’. It is then of key importance for our understanding of the evolution of lipocalins that YxeF is from the Gram-positive bacterium *B. subtilis*. It has been suggested that lipocalins first emerged along with the evolution of the outer membrane in Gram-negative bacteria [21]. The identification of distant homology between Blc and YxeF now suggests instead that lipocalins may have emerged before the divergence of Gram-positive and Gram-negative bacteria [28]. Although the presence of YxeF in *B. subtilis* might also have resulted from horizontal gene transfer, the genomic island predictor Alien_hunter [29] applied to genomic regions in and around the yxeF gene in both *B. subtilis* and *B. amyloliquefaciens* did not provide any support for this hypothesis. Hence, based on the evidence collected so far, it is thus indeed tempting to speculate that an ancient lipocalin, like protein YxeF devoid of a second hydrophobic core, evolved into both YxeF-like proteins of Gram-positive bacteria and the lipocalins present nowadays in Gram-negative bacteria and Eukaryotes. Additional structural variability of the β-barrel, required to convey specificity for other physiological ligands, might have co-evolved with the formation of the second hydrophobic core and, possibly, the disulfide bridges present in many eukaryotic lipocalins in order to maintain, or even increase protein stability [30].

As indicated above, the C-terminal domain of a self-compartmentalizing protease Pab87 from *Pyrococcus abyssi* has been suggested to be the first domain with lipocalin-like architecture from Archaea [17]. Notably, the Pab87 structure (i) ranks high among the top DALI structural matches for YxeF (Z-score 7.2; r.m.s.d. 2.8 Å, 76 aligned residues, 5% sequence identity), (ii) likewise represents a slim ‘β-barrel only’ structure, and (iii) contains a calycin signature motif (Gly-X-Tyr/Lys). However, the DALI Z-score for comparison of the β-barrels (Table 2) is comparably low, and the domain does not exhibit the ‘Ω-type’ loop characteristic of lipocalins. Consistently, its function was linked to protein oligomerization (and thus compartmentalization of the active site of the full-length protein) and not to the binding of a ligand in a fashion typically observed for lipocalins. Intriguingly, however, comparison of the structures of YxeF and the C-terminal domain of Pab87 indicates that possibly a very ancient lipocalin may have existed, even before the divergence of Bacteria and Archaea occurred.

### Prospects for Protein Design

The discovery of the ‘slim’ lipocalin YxeF devoid of the C-terminal α-helix (and of the entire second hydrophobic core) as well as disulfide bridges also provides a promising new scaffold for the design of lipocalins with novel ligand binding functions, so-called ‘anticalins’ [31]. The four structurally variable loops that form the entrance to the ligand pocket at the open end of a lipocalin’s β-barrel share functional similarity with the six hypervariable loops (CDRs) of antibodies. When compared with immunoglobulins, however, lipocalins are much smaller (∼160–180 residues), comprise only a single polypeptide chain, and can be produced at high yields in microbial host cells. Using targeted randomization of the structurally variable loop region in combination with phage display selection, anticalins with novel specificities have been engineered for the high affinity complexation of both low molecular weight compounds and protein antigens [7], [31], [32].

In many cases the lipocalin β-barrel is thermally rather stable and tolerates a wide range of amino acid substitutions at the ligand-binding site. Melting temperatures of natural lipocalins are often above 70°C [30] and can range beyond 95°C, for example, for human tear lipocalin. As mentioned above, Blc is actually a notable exception having a melting temperature of just ∼45°C. Attempts to engineer anticalins that lack the C-terminal α-helix were thus far not successful. Hence, the soluble domain of YxeF may turn out to be a promising target for the design of a novel line of minimal ‘β-barrel-only’, or ‘slim’ anticalins that can be used as reagents for bioanalytical purposes or separation tasks. It is evident that NMR and X-ray crystallographic studies [23], [33] will continue to be of key importance for this endeavor. Finally, since endogenous lipocalins are believed to play a role in antibiotic resistance and activation of immunity in Gram-negative bacteria, lipocalins of Gram-positive bacteria might turn out to be relevant biomedical targets themselves, *e.g.*, for the development of new antibiotics [24].

### Conclusions

The structure of the soluble domain of lipoprotein YxeF from the Gram-positive *B. subtilis* revealed an unexpected distant homology with lipocalin Blc from Gram-negative *E. coli*. Because YxeF is devoid of a second hydrophobic core typical for all lipocalins, we propose to introduce a new lipocalin sub-family named the ‘slim lipocalins’, with the members of Pfam family PF11631 being the first known representatives. The identification of YxeF as the first lipocalin homologue from a Gram-positive bacterium has far reaching consequences for our understanding of the evolution of this important class of proteins: lipocalins may have emerged well before the evolutionary divergence of Gram-positive and Gram-negative bacteria. Furthermore, we expect that the discovery of the ‘slim lipocalin’ YxeF will impact design of new anticalins with prescribed binding specificities. The results presented in this publication thus exemplify the role of structural genomics to generate new biological hypotheses and to support protein design efforts.

## Materials and Methods

### NMR Sample Preparation

The soluble domain of protein YxeF (excluding the ‘lipobox’ signal sequence) was cloned, expressed and purified following standard protocols developed by the NESG for production of uniformly *U*-^13^C,^15^N and 5%-^13^C, *U*-^15^N-labeled protein samples [34], [35]. Briefly, the truncated *yxeF* gene from *Bacillus subtilis* containing residues 19–144 was cloned into a pET21 (Novagen) derivative, yielding plasmid SR500A-21.2. The resulting construct contains eight nonnative residues at the C-terminus (LEHHHHHH) that facilitate protein purification. This expression vector, NESG SR500A-21.2, has been deposited in the PSI Materials Repository (http://psimr.asu.edu/). *E. coli* BL21 (DE3) pMGK cells, a codon enhanced strain, were transformed with SR500A-21.2 and cultured in MJ9 minimal medium containing (^15^NH_4_)_2_SO_4_ and *U*-^13^C-glucose or 5% *U*-^13^C-glucose/95% unlabeled glucose [36]. *U*-^13^C, ^15^N and 5%-^13^C, *U*-^15^N-labeled yxeF protein was purified using an AKTAxpress (GE Healthcare) based two-step protocol consisting of IMAC (HisTrap HP) and gel filtration (HiLoad 26/60 Superdex 75) chromatography. The final yield of purified *U*-^13^C, ^15^N and 5%-^13^C, *U*-^15^N protein YxeF (>98% homogenous by SDS-PAGE; 16.1 kDa by MALDI-TOF mass spectrometry) was ∼63 mg/L and ∼23 mg/L, respectively. In addition, *U*-^15^N, 5% biosynthetically directed fractionally ^13^C-labeled samples were generated to stereo-specifically assign Val and Leu methyl groups [37]. The final concentrations of *U*-^13^C, ^15^N and 5%-^13^C, *U*-^15^N labeled YxeF protein samples were about 1.1 mM and 1.2 mM, respectively, in a 90% H_2_O/10% D_2_O solution containing 100 mM NaCl, 5 mM CaCl_2_, 10 mM DTT, 0.02% NaN_3_, 50 µM 4,4-dimethyl-4-silapentane-1-sulfonic acid (DSS) and 20 mM 2-(N-morpholino)ethanesulfonic acid (MES) at pH = 6.5. An isotropic overall rotational correlation time of 8.6 ns was inferred from ^15^N spin relaxation times indicating that the protein yxeF is monomeric in solution. This conclusion was confirmed by analytic gel-filtration (Agilent Technologies) followed by a combination of static light scattering and refractive index (Wyatt Technology).

### NMR Spectroscopy

NMR spectra were recorded at 25°C on Varian INOVA 600 or 750 spectrometers equipped with cryogenic probes. Five through-bond correlated G-matrix Fourier transform (GFT) NMR experiments complemented by three-dimensional (3D) HNNCO as described [38]–[41], were collected for backbone and side chain resonance assignment (total measurement time: 81 hours). Simultaneous 3D ^15^N/^13^C^aliphatic^/^13^C^aromatic^-resolved [^1^H,^1^H] NOESY (nuclear Overhauser enhancement spectroscopy; mixing time: 60 ms; measurement time: 26 hours) was acquired to derive ^1^H–^1^H distance constraints [40]. Two-dimensional (2D) constant-time [^13^C,^1^H]-HSQC spectra were recorded as was described for the 5% fractionally ^13^C-labeled samples to obtain stereo-specific assignments for isopropyl groups of Val and Leu [41]. In order to assess thermal stability of protein YxeF, a series of 2D [^15^N,^1^H]-HSQC spectra were recorded for a ∼150 µM protein solution between 25°C and about 50°C on a Varian INOVA 500 spectrometer equipped with a conventional probe (total measurement time: 30 hours; Figure 1B). All spectra were processed and analyzed with the programs NMRPIPE and XEASY, respectively [42], [43].

Sequence specific backbone (^1^H^N^, ^15^N, ^1^H^α^, ^13^C^α^) and ^1^H^β^/^13^C^β^ resonance assignments were obtained by using (4,3)D HNNC ^αβ^ C ^α^/C ^αβ^ C ^α^(CO)NHN and (4,3)D H ^αβ^ C ^αβ^(CO)NHN along with the program AUTOASSIGN [44], and polypeptide backbone ^13^C’ resonances were assigned using 3D HNNCO. More peripheral side chain chemical shifts were assigned with aliphatic (4,3)D HCCH and 3D ^15^N/^13^C^aliphatic^/^13^C^aromatic^-resolved [^1^H,^1^H]-NOESY (for details of NESG NMR protocols, see http://www.nmr2.buffalo.edu/nesg.wiki). Overall, assignments were obtained for 99% of the backbone (excluding the N-terminal NH_3_^+^, the Pro ^15^N and the ^13^C’ preceding prolyl residues; Figure 1A) and ^13^C^β^, and for 98% of the side chain chemical shifts (excluding Lys NH_3_^+^, Arg NH_2_, OH, side chain ^13^C’ and aromatic ^13^C^γ^) which are assignable with the set of NMR experiments provided above. Furthermore, 79% of Val and Leu isopropyl moieties and 20% of β-methylene groups with non-degenerate proton chemical shifts were stereo-specifically assigned (Table 1). Chemical shifts were deposited in the BioMagResBank (accession code: 15211) [45]. ^1^H–^1^H upper distance limit constraints for structure calculations were extracted from NOESY (Table 1). In addition, backbone dihedral angle constraints were derived from chemical shifts using the program TALOS for residues located in well-defined secondary structure elements [46]. The programs CYANA and AUTOSTRUCTURE were used in parallel to assign long-range NOEs [47]–[50]. The final structure calculations were performed using CYANA followed by explicit water bath refinement using the program CNS [51]. NMR structure quality was assessed with the Protein Structure Validiation Software Suite (PSVS) and evaluated by structural genomics consortia, and RPF [52], [53]. The coordinates were deposited in the RCSB Protein Data Bank (PDB) with accession code 2JOZ [54]. Amino acid numbers in the PDB coordinate file are those of the soluble domain only, numbered as residues 2–127 (with residue 1 being the Met start residue of the recombinant protein). The residues of the soluble domain correspond in UniProt sequence P54945 to residues 19–144, which is the numbering used throughout the paper.

### Structural Bioinformatics

*In silico* studies of YxeF were primarily performed using the MarkUs server integrating a variety of computational tools [55], including the programs DALI and Skan for identification of structural similarities and calculation of structural alignments [16], [56], [57]. Moreover, the DALI pairwise alignment server was used to refine structural comparisons [58]. The programs MOLMOL [59] and STRIDE [4] were used to identify the location of regular secondary structure elements. A homology model was obtained for protein A7ZAF5, that is, one of the most distant known sequence homolog of YxeF, by submitting the YxeF-A7ZAF5 BLAST pairwise sequence alignment to the SWISS-MODEL server in alignment mode [60], [61]. Given the high sequence identity between template and target (61%) and the absence of any gaps, the comparative spatial localization of acidic residues inside the β-barrel appears to be robust. Alien_hunter genomic island predictions were obtained *via* the EnsemblBacteria website [29], [62].
